# Improvement of Free T4 in Newly Diagnosed Graves Disease Patients Through a Multifaceted Quality Improvement Approach

**DOI:** 10.1097/pq9.0000000000000824

**Published:** 2025-06-25

**Authors:** Einas H. Alkhatib, Tejal Patel, Julie Harlam, Padmaja Pavuluri, Maria Naveed, Andrew Dauber, Priya Vaidyanathan

**Affiliations:** From the * Division of Endocrinology/Diabetes, Department of Pediatrics, Children’s National Hospital, Washington, D.C.; † Division of Endocrinology/Diabetes, Department of Pediatrics, Boston Children’s Hospital, Boston, Mass.; ‡ Department of Pediatrics, Harvard Medical School, Boston, Mass.; § Division of Endocrinology/Diabetes, Department of Pediatrics, Goryeb Children’s Hospital/Atlantic Health System, Morristown, N.J.; ¶ Department of Pediatrics, George Washington University School of Medicine and Health Sciences, Washington, D.C.; ‖ Division of Hospital Medicine, Department of Pediatrics, Children’s National Hospital, Washington, D.C.; ** Division of Endocrinology/Diabetes, Department of Pediatrics, Children’s National Hospital, Washington, D.C.

## Abstract

**Introduction:**

Graves disease (GD) is the most common cause of pediatric hyperthyroidism, and if untreated, may result in multisystem complications and decreased quality of life. Through a multifaceted quality improvement (QI) approach, we aimed to address treatment barriers after a new diagnosis of GD and increase the percentage of patients attaining an euthyroid state within 3 months from diagnosis and sustain for 12 months.

**Methods:**

Using standard QI methodologies from January to November 2023, our plan, do, study, act cycles focused on an educational handout and checklist at diagnosis, a standardized methimazole dose based on initial free thyroxine (T4) and age, and frequent provider check-ins with phone call at 2 weeks, telehealth visit at 4 weeks with laboratories, and continued QI follow-up for 3 months as process measures. Outcome measure was the percentage of patients achieving normalization of free T4 level by 3 months. We used an electronic dashboard to track patients.

**Results:**

Of the 46 patients, 76% (34) received written education; 67% (30) were initiated on standardized methimazole dosing; 80% (37) and 70% (32), respectively, attended the 2-week telephone and 1-month telehealth visits, and 83% (38) obtained 1-month laboratories. By 3 months, the outcome measure increased to 78% (36/46) from 47% (15/32) (*P* < 0.01), sustained at 6 months (58% versus 22%) but decreased by 12 months (45% versus 40%). Barriers included missed appointments/laboratories, language, medication adherence, and/or lack of insurance.

**Conclusions:**

Through a multifaceted QI approach, we increased the percentage of newly diagnosed GD patients with normal free T4 levels and sustained for 6 months. Extension of follow-up is planned.

## INTRODUCTION

Although Graves disease (GD) is an overall rare condition among children, it is the most common cause of pediatric hyperthyroidism, affecting more women than men and occurring at any age with a peak in incidence during adolescence.^1^ Typical symptoms which should lead to suspicion of hyperthyroidism are unintentional weight loss, tachycardia, palpitations, heat intolerance, and hyperactivity.^1^ If hyperthyroidism is left untreated, it may lead to cardiomyopathy, heart failure, poor neuropsychological outcomes, or precipitate a thyroid storm.^1,2^ Thyroid storm is a life-threatening condition that can be fatal. It can be precipitated by severe illness, undiagnosed GD, or GD with poor compliance to treatment.^2^

GD is an autoimmune disorder diagnosed by suppressed thyroid-stimulating hormone (TSH), elevated thyroid hormone levels (free thyroxine [free T4] and triiodothyronine [T3]), and elevated antibody levels of thyroid-stimulating immunoglobulin.^1^ Medical therapy with antithyroid medication, specifically methimazole, and adjuvant beta-blocker treatment is the currently the first line therapy for GD in children. The recommended starting dose of methimazole is variable, ranging from 0.25 to 1.0 mg/kg/d divided BID, with a maximum of 30 mg/d.^1,2^ Methimazole targets the reduction of thyroid hormone synthesis and aids in remitting the underlying autoimmune process.^2^ Methimazole treatment may continue for several years to see if remission can be achieved. Definitive treatment with either thyroidectomy or radioactive iodine ablation is pursued if there are complications from medical therapy, lack of remission after prolonged medical therapy, or due to patient preference.^1,2^

Although uncontrolled disease and symptoms can negatively impact the quality of life,^3^ there is no literature that outlines the optimal time when euthyroid state should be achieved after diagnosis. Current practices suggest obtaining labs every 4–6 weeks and adjusting methimazole doses to achieve normalization of thyroid levels.^2^ Quick normalization to euthyroid state would allow for rapid resolution of symptoms, improved quality of life, and mitigate symptoms. Through review of our institutional electronic database, we determined that our baseline percent of patients attaining a normal free T4 within 3 months was only 47%. Through a multifaceted quality improvement (QI) approach, we aimed to address treatment barriers after a new diagnosis of GD and to improve percentage of patients attaining an euthyroid state (defined as a normal FT4) from a baseline of 47% to 85% within 3 months from diagnosis and to sustain this for 12 months by addressing treatment barriers.

## METHODS

An electronic thyroid dashboard was created with our Information Technology department to monitor new GD patients with columns for patient name, date of diagnosis, sex, race, ethnicity, and if/when a normal FT4 level was attained within 3 months. Patients were automatically listed on the dashboard if they received a diagnostic code for GD or Hyperthyroidism. If patients coded for Hyperthyroidism did not meet criteria for GD, the QI team manually excluded them from the dashboard. The QI team consisted of 2 endocrinology fellow physicians, an NP, and an attending physician. This QI initiative was exempt from requiring institutional review board approval. The initiative was implemented in a pediatric endocrinology clinic within a large, academic children’s hospital in Washington, D.C.

**Fig. 1.**
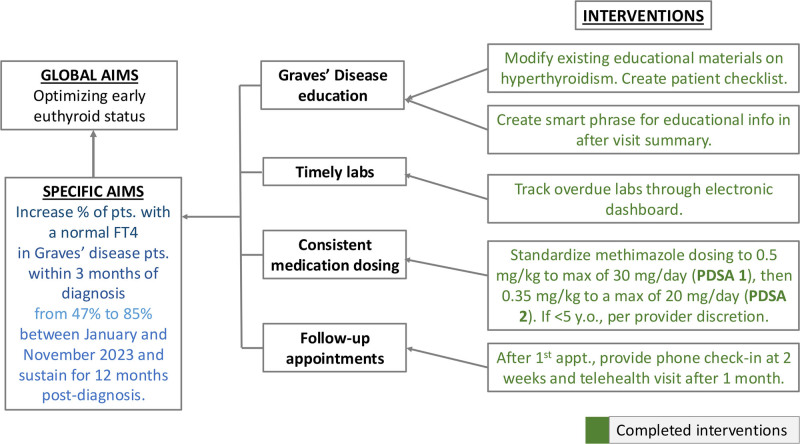
Key driver diagram of GD multifaceted QI approach for PDSA 1–2.

We assessed baseline data from 32 patients with GD diagnosed from January to December 2022. Using QI methodologies, including a key driver diagram (Fig. 1) and multidisciplinary input from our division, we identified disease education, language, lack of standardized medication dosing, and timely reassessments as barriers to attaining a normal FT4 within 3 months and designed our first plan, do, study, act (PDSA) cycle to address these.

The QI interventions were from January 2023 to November 2023. Subsequently, a chart review was conducted at 6 and 12 months postdiagnosis to obtain additional data.

### PDSA Cycle 1

**Fig. 2.**
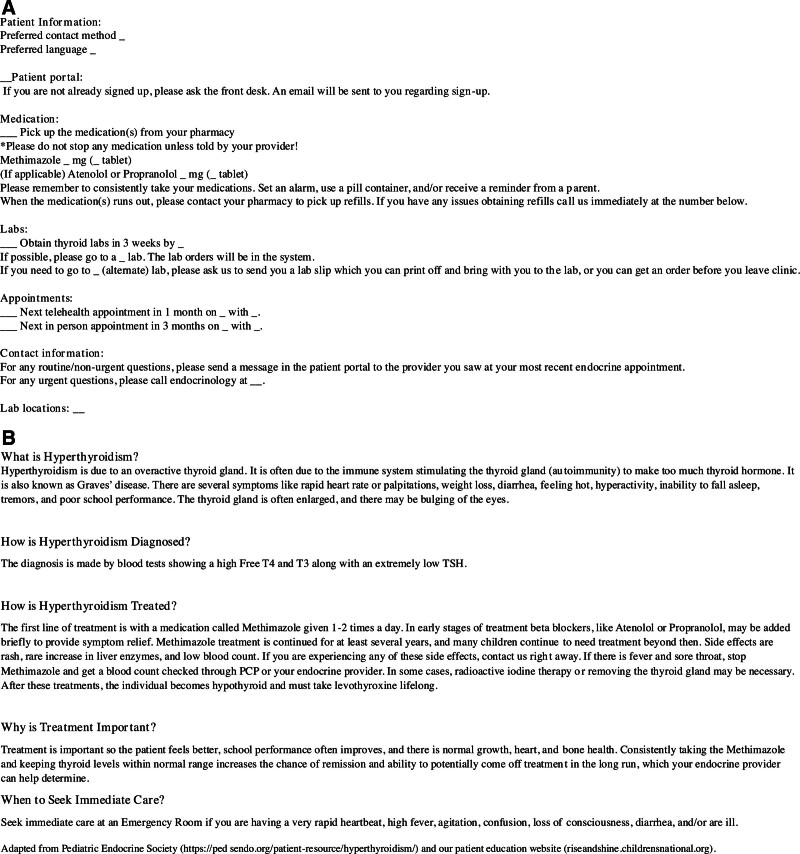
GD AVS. A, Patient checklist. B, Patient handout.

Our first PDSA cycle was from January to June 2023 and included introducing a comprehensive bundle: (1) patient checklist (Fig. 2A) filled out by the provider, and an educational handout^4,5^ (Fig. 2B), both created as an EMR smart phrase and given to patients as an After Visit Summary (AVS) in English or Spanish at all initial GD appointments; (2) standardized methimazole dosing; and (3) close follow-up.

We implemented a standardized methimazole dosing at 0.5 mg/kg with a maximum of 30 mg daily, for patients older than 5 years of age, and for patients younger than 5 years of age, methimazole dosing was left to the provider’s discretion, as was the need for beta blockers (atenolol or propranolol) and dosing for patients of all ages. Dosing frequency was per provider discretion; twice daily was more common than once daily. Adherence to dosing was defined as within 0.1 mg/kg of the recommended dose; rounding of doses was sometimes necessary based on the available milligram tablet options.

At the initial clinic visit, a baseline TSH, free T4, total T3, thyroid-stimulating immunoglobulin, complete blood cell count, and comprehensive metabolic panel were obtained. We counseled all patients on potential methimazole side effects, including rash, arthralgias, fever, sore throat (risk for agranulocytosis), and jaundice. A language interpreter was present via video at the appointments when necessary.

After the initial visit, we added a 2-week telephone check-in with a nurse practitioner (NP) from our QI team reminding patients to take medications, check on symptoms, side effects, future follow-up laboratories, and appointments. Patients then had a 1-month telehealth visit with the same NP to review the 1-month laboratories. Methimazole dose was adjusted based on FT4 levels and most discontinued beta blockers at that time. Methimazole dosing was increased if the FT4 was >2, despite consistent compliance, and dosing was decreased if the FT4 was lower than the normal reference range. We did not see a significant difference in total T3 versus free T4 values and therefore relied on free T4 levels for adjustments. If methimazole dose was adjusted, additional labs were obtained every 3–4 weeks based on trend until the 3-month visit with an endocrinology provider in the division.

### PDSA Cycle 2

We implemented the second PDSA cycle from July to November 2023. First, this included a reduction to the standardized methimazole dosing for patients older than 5 years of age and an initial FT4 < 4, to 0.35 mg/kg/d with a maximum of 20 mg/d. For patients older than 5 years of age, with initial FT4 ≥ 4, the dosing remained at 0.5 mg/kg/d with a maximum of 30 mg. The dose was reduced to minimize hypothyroidism at the follow-up laboratories. Second, we also changed the laboratory follow-up period to 3 weeks instead of 4 to ensure availability of results at the 4-week telemedicine visit, and third, added a list of local laboratory addresses, office hours, and contact information to aid in obtaining labs on time.

### Postintervention

Chart reviews were conducted at 6 and 12 months postdiagnosis. Barriers to sustaining a normal FT4 were determined by chart review.

### Data Analysis

**Fig. 3.**
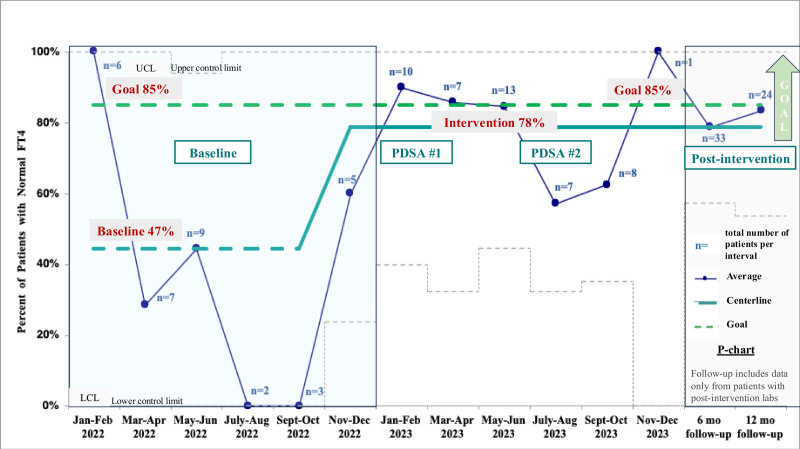
P-chart: Percentage of patients with normalization of free T4 within 3 months of GD diagnosis for baseline (January–December 2022) and intervention groups (January–November 2023), as well as percent with sustained normal free T4 at 6 and 12 months postdiagnosis.

Data were collected for one year before and during QI implementation, as well as postintervention. Electronic data were securely stored, and patient confidentiality was maintained. Statistical analysis of subgroups included Fisher exact test.^6^ *T* test and weighted percentages were used to compare compliance to baseline and QI initiative components. Outcome data are displayed on a p-chart (Fig. 3; Tables 1, 2.

**Table 1.** Demographics for Baseline and QI Initiative Groups

	Baseline (n = 32)	Intervention (n = 46)
Age, y (mean ± SD)	12 ± 5	13 ± 4
Sex		
Female	84%	83%
Male	16%	17%
Race		
White	6%	6.5%
Black	28%	30%
Asian	6%	6.5%
Mixed	35%	24%
Other/unknown	25%	33%
Ethnicity		
Hispanic/Latino	44%	39%
Non-Hispanic/Latino	53%	61%

**Table 2.** QI Metrics for Baseline and Initiative Groups

	Baseline (n = 32)	Intervention (n = 46)
Initial FT4, ng/dL	4.43 ± 2.73	5.38 ± 2.74
AVS received	45%	76%
Standardized methimazole dose	30%	67%
Provider check-in		
2 wk	0%	80%
1 mo	0%	70%
Laboratories obtained postdiagnosis		
1 mo	70%	83%
6 mo	50%	74%
12 mo	53%	55%
Normal FT4 from time of diagnosis		
3 mo*	47%*	78%*
6 mo†	44%	79%
6 mo‡	22%	58%
12 mo†	76%	83%
12 mo‡	40%	45%

* *P* value < 0.001.

† Patients with available labs at 6 months (n = 16/32 baseline, n = 33/46 intervention) and 12 months (n = 17/32 baseline, n = 24/46 intervention).

‡ All patients at 6 and 12 months (n = 32 baseline, n = 46 intervention).

## RESULTS

Demographics, methimazole dose, and adherence to QI initiatives and outcome measures of the 32 baseline patients and 46 QI patients are in Tables 1, 2. The initial methimazole doses in the baseline group were not standardized and ranged between 0.1 and 0.75 mg/kg/d (Table 2).

By 3 months, 78% of patients (36/46) in the QI initiative group attained a normal FT4 level, compared with 47% at baseline (Fig. 3, p-chart) (*P* < 0.001).

Methimazole doses were overall well tolerated with no side effects in 78% of patients, whereas 16% experienced a rash and 6% developed itching. Of those with symptoms, two-thirds were in PDSA 1, whereas one-third were in PDSA 2; however, this was not a statistically significant difference. Symptoms improved with cetirizine and/or transient dosing decrease. None had to come off methimazole permanently.

After 1-month follow-up laboratories, 30% of patients were continued on the same dose, 23% had a dose increase, and 47% had a dose decrease (7% had a FT4 < 0.7 and/or elevated TSH). Of the patients with a normal FT4 level at 1 month, their average initial methimazole dose was 0.4 mg/kg.

For the 10 patients who did not reach a normal FT4 within 3 months, barriers identified through discussions with patients and chart review included largely missed appointments and/or laboratories, suboptimal medication adherence, and to some extent, non-English speaking patients found it a little harder to keep up with the process, and a few lost medical insurance coverages in this time period.

Outcomes at 6 and 12 months postanalysis for patients with available laboratory data are shown in Figure 3. Due to transferring care, 1 patient was excluded at 6 months and another at 12 months. Although 58% had normal free T4 at 6 months, only 45% had normal free T4 at 12 months, and the drop off in these outcomes was due to not having consistent follow-up laboratories. A subanalysis of patients who had consistent labs showed 79% (26/33) were euthyroid at 6 months and 83% (20/24) were euthyroid at 12 months. Further comparison between the baseline and QI initiative groups is in Table 2.

## DISCUSSION

To conclude, a multifaceted QI approach of an AVS consisting of a patient checklist and educational handouts, standardized methimazole dosing, and consistent check-in, and laboratory draws with methimazole dose adjustments led to a significant increase in our newly diagnosed patients with GD having a normal FT4 (47%–78%) within 3 months.

Our QI design targeted comprehensive aspects of patient care, including an electronic dashboard to track patient diagnosis and outcomes, patient-centered education, medication dosing, consistent follow-up with a designated provider after the initial visit, along with periodic QI team discussions to review outcomes, as well as share QI protocol reminders to providers. Although previous thyroid QI studies primarily focused on the use of electronic dashboards,^7^ this QI design included a multifaceted patient-centric approach, which can feasibly be followed at other sites even in the absence of an electronic dashboard. As language can be a significant barrier to patient education, we included written AVS documents in Spanish and had interpreters available for patient encounters. The standardized medication dose was well tolerated. Although our rate of transient rashes (16%) was slightly higher than prior studies (8% on 0.3 mg/kg),^8^ medication compliance was not affected. None of our patients developed agranulocytosis.

There are a few limitations in this study. Our sample size is small, and we may need a few additional cycles of this QI initiative to build a larger patient cohort to further validate the approach. Provider adherence to AVS use (45% baseline to 76%) was limited by time in a busy clinical practice. It would also be important to survey providers on barriers to using standardized dosing. Although patients did well during the QI initiative and up to 6 months from diagnosis, there was no difference overall in percentage attaining a normal FT4 at 12 months between the baseline and QI group. Those who had consistent labs in both groups were able to achieve comparable FT4 results (76% in the baseline group versus 80% in the QI initiative group). This underscores the importance of consistent laboratory monitoring and follow-up to achieve disease control and further supports that in chronic disorders like GD, it is important to continue intensive monitoring indefinitely for optimal outcome. Despite the QI measures, some patients failed to achieve a normal FT4 within 3 months. Through chart review by the QI team and discussions with patients, we identified delay in getting labs on time as a major barrier, despite reminders and medication adherence. Despite the availability of interpretation services, language barriers prevented some families from promptly reaching out to the providers with concerns. Lack of and/or lapse of insurance prevented or delayed patients from obtaining labs or filling prescriptions. Our future PDSA cycles will further address these barriers by providing a pill organizer for medication reminders, 1-month in-person visits, instead of telemedicine visits, for those who have technology and/or language barriers, and social work support for patients with missed appointments, laboratory draws, or insurance concerns.

There was some variability in the percentage of patients attaining normal FT4 during the summer months. Patients may have less structured schedule regimens than during the school year, which may indicate the importance of even closer provider check-ins during these months. Although a formal QoL scale such as PedsQL Generic Core Scales^3^ was not utilized, this can be incorporated in the future, especially for evaluation of school performance to see if there is a difference in academic performance in patients achieving normal FT4 by 3 months of diagnosis.

Our QI project led to a significant improvement in the percent of patients attaining a normal FT4 within 3 months. We plan to extend our intervention through the first few years of diagnosis for long-term sustained results. Finally, we anticipate that this QI model could be applied to other chronic conditions, beyond new onset GD.

## ACKNOWLEDGMENT

The authors thank the Children’s National Information Technology department for creation of the hyperthyroidism electronic dashboard.

## ETHICAL APPROVAL

This article was written ethically in accordance with the World Medical Association Declaration of Helsinki. It was exempt by the institutional review board.
